# Exploring Noncentrifugal Sugar as a Partial Replacement for White Sugar in Low Methoxyl Pectin Confectionery Gels: Impacts on Physical and Rheological Properties

**DOI:** 10.1021/acsfoodscitech.4c00603

**Published:** 2024-12-10

**Authors:** Hafiz Imran Fakhar, Elif Cavdaroglu, Muhammad Qasim Hayat, Hussnain A. Janjua, Mecit Halil Oztop

**Affiliations:** † Medicinal Plant Research Laboratory, Department of Agricultural Sciences and Technology, Atta-ur-Rahman School of Applied Biosciences (ASAB), National University of Sciences & Technology (NUST), Sector H-12, Islamabad 44000, Pakistan; ‡ Department of Microbiology & Biotechnology, Atta-ur-Rahman School of Applied Biosciences (ASAB), National University of Sciences & Technology (NUST), Sector H-12, Islamabad 44000, Pakistan; § Department of Food Engineering, Middle East Technical University (METU), Ankara 06800, Turkey; ∥ Department of Food Engineering, Izmir Institute of Technology, Izmir 35430, Turkey

**Keywords:** Noncentrifugal sugar, low-methoxyl pectin, gelation, NMR, texture

## Abstract

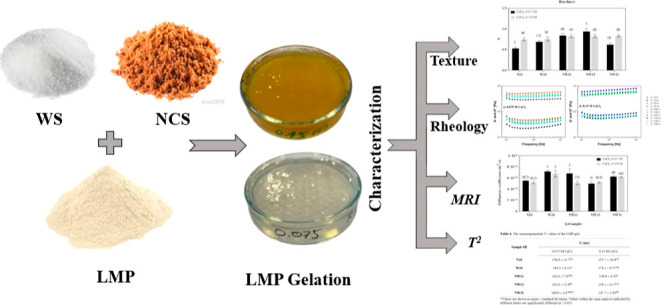

Noncentrifugal sugar (NCS) is an unrefined, dark brown sugar containing minerals and plant secondary metabolites, unlike refined white sugar (WS). This study explored using NCS in confectionary jellies as an alternative sugar. We used different concentrations of NCS and WS to prepare low methoxyl pectin (LMP) confectionery gels characterized by their physical and rheological properties along with time-domain nuclear magnetic resonance (TD-NMR) relaxometry. The strongest LMP gel, with a hardness of 0.94 N, was achieved by substituting 25% of WS with NCS at a low CaCl_2_ concentration (0.075 M). Gels with up to 50% WS replaced by NCS showed comparable hardness to standard LMP gels made solely with WS at a 0.15 M CaCl_2_ concentration, attributed to NCS’s unique constituents. The NCS–WS gel exhibited the shortest T_2_ values (139.8 ms) and self-diffusion coefficient values (4.99 × 10–10 m^2^/s), indicating a denser, more cross-linked structure that restricted water mobility. These findings suggest NCS’s complex role in affecting LMP gels’ chemical and physical properties, highlighting its potential as a partial WS replacement in LMP gelation-based products, with an additional source of minerals and antioxidants.

## 1 Introduction

Noncentrifugal sugar (NCS) is the solid cane sugar obtained through the evaporation of sugar cane juice, which undergoes minimal or no refining. It is also known by various names worldwide, such as panela in Latin America, jaggery in India, and kokuto in Japan.^1,2^ NCS can be considered a nutraceutical and functional ingredient due to its rich content of minerals, bioactive compounds, flavonoids, and phenolic acids, which have been shown to exhibit antioxidant and anti-inflammatory properties.^3,4^ Substituting the consumption of refined white sugar (WS) with NCS may help to manage chronic diseases commonly associated with obesity, diabetics, neurodegeneration, oxidative stress, and inflammation.^5−9^ The production process for NCS typically involves harvesting the sugar cane, washing, and crushing the plants to extract the juice, and then boiling the juice to concentrate and crystallize the sugar. Unlike WS, NCS does not undergo additional processing steps such as bleaching, filtering, and chemical treatment to remove impurities and color. As a result, NCS does not treat with industrial additives such as sulfur dioxide, phosphoric acid, calcium hydroxide, and activated carbon instead; it retains more of the natural plant matter, such as minerals and vitamins, that are present in the original juice.^10^

NCS is naturally high in moisture and has a darker color due to the high molasses content (3–7%, w/w) and the stronger flavor than WS. It is often used as a natural sweetener in baking, cooking, and beverages and is also a popular ingredient in natural and organic food products.^5^ Thus, NCS can be a good alternative to replace WS in sugar-based foods, such as jams and jellies. Previously,^11^ used NCS in strawberry and kiwifruit jams instead of WS and examined the physicochemical, antioxidant, microbiological, and sensory properties. They detected no significant changes in physicochemical properties except for color values but observed a higher antioxidant capacity with the addition of NCS. Sensory panels also showed good acceptance with the replacement of NCS.

Jelly confectioneries are prepared using different gelling agents such as gelatin, starch, pectin, and carrageenan.^12,13^ Primarily, vegan and “halal” requirements of the Muslim community shifted industry to produce higher volumes of plant-based confectionaries. Unlike high methoxyl pectin (HMP), which requires high amounts of sugar (>55%, w/w) to form a gel, low methoxyl pectin (LMP) can gel at low sugar concentrations (30%, w/w) through ionic linkages via calcium bridges between carboxyl groups of two different chains over a wide range of pH making it an attractive alternative for creating low-calorie confectionery products.^14^

The gelation process of pectin is highly specific and influenced by the degree of methoxylation of the pectin, allowing for the formation of gels under varying pH and sugar concentration conditions. In the presence of sugar, LMP gelation occurs through a combination of two-stage mechanisms. In the first stage, the pectin chains are associated with the formation of dimers, which are driven by the electrostatic interactions between the calcium ions and carboxylate groups of pectin. In the second stage, aggregation of dimers occurs with the formation of junction zones.^15^ The stability of the junctions depends on the strength of the electrostatic bonds. Under acidic conditions, galacturonic acid units protonate, revealing less dissociated carboxylic groups for cross-linking with Ca^2+^. Thus, hydrophobic interactions, hydrogen bonds, and the number of charged groups interacting with the pectin molecules take part in strengthening the gel formation of LMP.^16^ Sugar promotes gelation at this point due to both increased hydrophobicity by decreasing water activity and creating hydrogen bonds between two pectin chains.^15^ The type of sugar and constituents in it may have an impact on LMP gelation. Therefore, it is hypothesized that both NCS and impurities presented in the NCS could affect the calcium-pectin interaction, resulting in gel properties different than WS.

Recently, Guo et al.^17^ have successfully demonstrated the formation of LMP gels with sugar cane molasses without the need for additional calcium and highlighted the inherent gelation properties of endogenous calcium ions found in that sugar. However, the current investigation focused on a distinct approach: utilizing NCS as the primary sugar source for LMP gelation. While both sugar cane molasses and NCS share common characteristics, such as their unrefined nature and the presence of impurities and minerals, they offer unique attributes to the gelation process. Sugar cane molasses is a byproduct of refined sugar extraction where sugar crystals are typically separated through a process known as centrifugation, leaving behind molasses, which is a thick, dark, syrupy residue.^18^ In contrast, NCS has not undergone the traditional centrifugal refining process of producing white sugar that is obtained directly from sugar cane juice. They serve different purposes in various applications and can have distinct properties compared to highly refined white sugar.

The high demands of vegan and “halal” requirements of the Muslim community shifted industry to produce higher volumes of plant-based confectionaries. So, NCS may be an attractive alternative for creating LM pectin-based low-calorie confectionery products, which may have additional nutritional values. Thus, this study aimed to explore the NCS effect in the replacement of WS in LMP gelation with respect to the textural and rheological properties of the gels. The influence of Ca^2+^ concentration on gel properties was also investigated. Time domain nuclear magnetic resonance (NMR) relaxometry was also used as an alternative method for the characterization of NCS containing LMP gels.

## 2 Materials and Methods

### 2.1 Materials

LMP with a DE of 27% and >65% galacturonic acid content was kindly provided by Cargill (Balikesir, Turkey). WS (Keskinkılıç Gıda Sanayi ve Ticaret A.S., Gebze, Kocaeli, Turkey) was purchased from a grocery store market in Ankara, Turkey. NCS was used for sugar replacement and was kindly provided by a local producer in Punjab, Pakistan, in triplicate. All the other chemicals were purchased from Sigma-Aldrich Chemical Co. (St. Louis, MO, USA). Distilled water was used for all the experiments.

### 2.2 Sugar and Minerals Analysis of NCS

The sugar analysis was performed by using an HPLC system consisting of an inertsil NH_2_ column (250 × 4.6, 5 μm, Shimadzu Sci. Ins., Kyoto, Japan), degasser, pump, autosampler, column oven, and refractive index detector. The mixture of acetonitrile and water with a ratio of 80:20 (v/v) was used as the mobile phase. Flow rate, injection volume, and oven temperature were 1 mL/min, 20 μL, and 50 °C, respectively. The calibration curve was prepared by each sugar solution in the range of 5–200 g/L. The mixture of sugar samples and water was prepared with a ratio of 1:10 and was filtered by using 0.45 μm nylon filters (PA).

The minerals analysis was performed using an inductively coupled plasma optical emission spectrometer (PerkinElmer Optima 4300 DV–ICP-OES).

### 2.3 Low-Methoxyl Pectin Gelation

Gels were prepared using 1% (w/w) LMP and 30% sugar (w/w), as described by the Food Chemicals Codex (1972).^19^ Two concentrations of CaCl_2_ (0.075 and 0.15 M) and different NCS to WS ratios (1:1, 1:3, and 3:1) were used. The NCS to WS ratio was selected based on the preliminary research that suggests these ratios are commonly used or theoretically significant in similar food product formulations.^20,21^ Samples were named based on the different ratios of sugars (Table 1). The control gels were prepared by the addition of WS and NCS only. In the low ester pectin jelly preparation, 0.3 g of pectin was mixed with 2 g of sugar in a beaker. A 21.25 mL citrate buffer (pH 2.8) was added to the beaker and mixed until it was dispersed. The solution was then heated to a boil with continuous stirring. A 7 g sugar mixture was added to the solution and continued boiling until it was dissolved. Then, 1.25 mL of 0.075 or 0.15 M CaCl_2_ solution was added while stirring until the net weight of 30 g was reached. The solution was allowed to set for around 1 h at room temperature and then stored for 18–24 h at 25 °C by wrapping the surface with aluminum foil until the testing. To prevent microbial activity in the gel, 0.01% (w/w) sodium azide was added. All of the gels were cylindrical in shape with a diameter of 50 mm and a width of 12 mm. Each gel was prepared in triplicate independently.

**Table 1:** LMP Gel Formulations Based on the Different Ratio of Sugars

sample ID	NCS (%, w/w)	WS (%, w/w)	Sugar ratio (NCS/WS)
N_10_	30	0	1:0
W_10_	0	30	0:1
NW_11_	15	15	1:1
NW_13_	7.5	22.5	1:3
NW_31_	22.5	7.5	3:1

### 2.4 Measurement of pH and Total Soluble Solids

The pH values of the gels were measured using a pH meter (Orion Star A211, Thermo Electron Corporation, Beverly, MA, USA) at room temperature. Brix measurements were performed by using a digital refractometer (HANNA, Cluj, Romania). All of the measurements were performed in triplicate independently.

### 2.5 Textural Analysis

The hardness of the gels was measured by using a Texture Analyzer (Brookfield Ametek CT3, TA10 probe, Middleboro, MA, USA) 1 day after gel preparation. A cylinder-shaped probe with a diameter of 17.5 mm and a load cell of 0.05 N was attached to the instrument to measure the hardness. Samples were compressed twice with a 50 mm/s pretest speed. The extension distance was adjusted to 0.68 cm. NEXIGEN texture analysis software was used for data analysis.^20,21^ All the measurements were performed in triplicate independently.

### 2.6 Rheological Measurements

Dynamic rheological measurements of all gels were conducted with a parallel plate rheometer (Kinexus Dynamic Rheometer, Malvern, Worcestershire, U.K.). 10 g of gel was used to measure the rheology, which was enough to fill the space of paralleled plates. A frequency sweep test was performed to measure storage modulus (*G*′, Pa), loss modulus (*G*″, Pa), and complex modulus (G*, Pa) over the range of 1 to 10 Hz with 1% oscillation strain at 25 °C.^22^ The rheological experiments were conducted in three replicates independently, and their average results were computed. The *G*′ and *G*″ data were fitted to the power-law model. The model equations are represented in eqs 1 and 2.
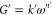
1
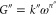
2where *k*′ and *k*″ are power law constants (Pa·sn′ and Pa·sn′′, respectively), and *n*′ and *n*′′ are referred to as frequency indices of storage and loss moduli (both dimensionless), respectively. ω is the angular frequency.

The strength of gels was estimated by fitting the Power law model on the complex modulus (G*) data, which was determined by eq 3.^23−25^
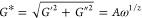
3*A* is a constant representing the forces between the flow units and accounts for the gel’s three-dimensional network structure; *z* is an index that accounts for the number of nodes and strands in a three-dimensional network.

### 2.7 TD NMR Relaxometry Experiments

TD NMR relaxometry experiments were conducted by using a 0.5 T (20.34 MHz) system (Spin Track, Resonance Systems GmbH, Kirchheim/Teck, Germany). For *T*_2_ measurements, the Carr–Purcell–Meiboom–Gill sequence was used with parameters of 2000 μs echo time, 700 echoes, and 4 scans.^21,26^ The measurements were performed for all samples in triplicate. The *T*_2_ data were analyzed by fitting the data to a monoexponential model conducted on the relaxation curves using MATLAB (Mathworks Inc., Version 2022a, U.S.A.).

### 2.8 Self-Diffusion Coefficient Measurements

A benchtop MRI system (Pure Devices GmbH, Germany) operating at the ^1^H frequency of 24.15 MHz, equipped with a gradient amplifier (Grad *x*: max 1.229 in *T*/*m*, Grad *y*: max 1.230 in *T*/*m*, and Grad *z*: max 1.515 in *T*/*m*) and a rf coil of 10 mm, was used. Diffusion readings were taken using a pulsed gradient spin echo (PGSE) sequence with parameters of 6.0 × 10^–10^ m^2^s^–1^ estimated diffusion, 20 ms echo time, and 550 ms longitudinal relaxation time. The measurements were performed for all samples in triplicate.

### 2.9 Statistical Analysis

Analysis of variance (ANOVA) was used to assess significant differences (*p* < 0.05) between the samples, and the Tukey test was used as the multiple comparison test using Minitab (ver.16.2.0.0, Minitab Inc., United Kingdom).

## 3 Results and Discussion

### 3.1 Sugar and Mineral Content of NCS

The sugar composition of NCS is listed in Table 2. Results showed that NCS had the highest sucrose content (88.57 ± 0.016%, w/w), followed by glucose (6.16 ± 0.001%, w/w) and fructose (4.43 ± 0.001%, w/w). These findings were consistent with those of ref (10) who reported the sucrose range in NCS as 76.5–89.5%. The glucose content was consistent with previously reported results, but the contents of glucose and fructose in this study were found to be higher than previously reported results.^3,27^

**Table 2:** Percentage of Sugars in NCS^a^

sugars	percentage of sugar (%)
sucrose	88.6 ± 1.63
glucose	6.16 ± 0.13
fructose	4.43 ± 0.05

a *Data are shown as mean ± standard deviation.

The composition of minerals in NCS used in this study is shown in Table 3. The highest composition of potassium (16,000 mg/kg) was found, followed by sulfur and sodium (3700 and 2500 mg/kg, respectively). The composition of magnesium and calcium appeared to be 700 mg/kg. The trace amount of aluminum and iron was also found in NCS. The composition of minerals was consistent with previous reports except for sulfur.^3,5,10,27,28^ Furthermore, the composition of minerals also varies based on the variety of sugar cane, soil type, fertilization strategies, climatic conditions, type of harvest, cutting and processing, and locality.^29^ The unique composition of sugars and minerals in NCS significantly affects the gelation process of low methoxyl pectin, influencing the gel’s texture and stability. Therefore, different batches of NCS can have different effects on the gel texture, and the results of this study refer to this composition.

**Table 3:** Composition of Minerals in NCS

minerals	amount (mg/kg)
Na	2500
Mg	700
Si	300
P	700
S	3700
K	16,000
Ca	700
Al	57
Fe	55

### 3.2 Effect of NCS Addition on the Total Soluble Solids (Brix) and pH of LMP Gels

The total soluble solids and pH of the LMP gels are shown in Table 4. The process of pectin gelation is complex, involving a combination of ionic and other interactions, as explored in numerous studies.^14,30−38^ Environmental factors such as pH, Ca^2+^, and soluble solids like sugar significantly influence the gelling process of various pectin types, often in contrasting ways.^37^ In this study, the pH of pectin gels was slightly below the p*K*_a_ of pectin (3.50)^14,15^ when WS was included in gel formulation with the lowest pH observed in the sample with the highest WS concentration (W_10_) at both Ca^2+^ concentrations. Conversely, the highest pH values were recorded in NCS with the N_10_ sample. Below the p*K*_a_, fewer carboxyl groups on pectin dissociate, limiting the formation of a stable egg-box structure.^33,39^

**Table 4:** pH and °Brix Values of LMP Gels^a^

sample ID	pH		°Brix	
	0.075 M CaCl_2_	0.15 M CaCl_2_	0.075 M CaCl_2_	0.15 M CaCl_2_
N_10_	3.52 ± 0.04^BC^	3.64 ± 0.04^A^	34.9 ± 0.71^BC^	33.4 ± 0.38^E^
W_10_	3.03 ± 0.04^G^	3.15 ± 0.03^F^	35.9 ± 0.44^A^	35.2 ± 0.80^AB^
NW_11_	3.48 ± 0.03^CD^	3.43 ± 0.03^D^	34.1 ± 0.35^DE^	34.7 ± 0.34^BCD^
NW_13_	3.27 ± 0.04^E^	3.17 ± 0.03^F^	34.1 ± 0.51^DE^	34.4 ± 0.39^CD^
NW_31_	3.56 ± 0.02^B^	3.58 ± 0.02^AB^	34.3 ± 0.36^CD^	34.3 ± 0.38^CD^

a *Values are shown as mean ± standard deviation. Values within the same analysis indicated by different letters are significantly different (*p* ≤ 0.05).

LMP gelation is facilitated at pHs higher than the p*K*_a_ of pectin since carboxyl groups need to be unprotonated to form Ca-bridges.^14,15^ Consequently, the formation of typical egg-box junction zones is expected to be limited in WS samples, and more interactions between undissociated carboxyl groups via hydrogen bonds would be formed instead. The WS content in the gels could promote hydrogen bonds with water molecules and then immobilize free water leading to a concentrated polymer environment for gelation to support the interchain interdimer associations as shown similarly to those reported in studies by^16^ and.^40^ The water-binding ability of WS, coupled with a small amount of calcium, is hypothesized to lead to successful ionic junctions during gelation, as described in previous studies by.^14,31,36^

Adding NCS to LMP gels significantly increased the pH of the gel (*p* < 0.05). This increase can be attributed to a combination of factors, including the presence of calcium or other minerals, polyphenols, and the ionization level of low methoxy (LM) pectin. The presence of alkaline minerals such as magnesium, phosphorus, and potassium in the range of 700–16,000 mg/kg, as shown in Table 3, and other impurities such as polyphenolic compounds in NCS.^10,27,41,42^ These impurities are a natural part of the NCS production process and can vary depending on the source and processing methods used to make the NCS. The alkaline minerals release hydroxide ions (OH^–^) upon dissolution in water, consequently increasing the solution’s alkalinity and potentially contributing to the observed pH increase in the LMP gel system. Depending on their chemical properties, polyphenols can act as weak acids or bases. Some polyphenolics might have a basic character, which could contribute to the increased pH.^43^ The addition of NCS, with its alkaline minerals and other constituents, might shift the ionization equilibrium, leading to an increase in the dissociation of −COOH groups to carboxylate ions (−COÔ–) and, consequently, an increase in pH.^16,37,44^ Furthermore, it was observed that the pH of the gels increased with the increase in CaCl_2_ concertation in N_10_ and W_10_ gels. Since Ca^2+^ is one of the alkaline minerals, it might increase the pH of the solution when it is dissolved in a gel. This can be attributed to the binding of Ca^2+^ to the −COOH groups, which subsequently releases −OH and causes a slight increase in the pH of the solution.

The Brix values also changed in a narrow range between 33.4 and 35.9 (Table 4). Replacing the WS with NCS showed a significant decrease in Brix values of gels (*p* < 0.05). Since NCS contained less sucrose and additional nonsucrose components, as shown in Table 2, the Brix value of N10 gel was found to be lower than that of W_10_, which was primarily composed of sucrose.^10^ Increased CaCl_2_ did not significantly change the Brix values of gels except for N_10_ (*p* > 0.05).

The function of sugar in the LMP gels is to bind water and promote interactions between the neighbored molecules.^37^ NCS, like WS, can serve as a water-binding agent in the gel by holding the water molecules to create a stable gel structure. However, the water binding capacity of NCS may vary compared to WS due to compounds other than sucrose in NCS, such as phenolics and minerals. These other compounds can introduce additional interactions within the gel matrix and affect the gel’s texture and strength, which will be discussed in further sections.

### 3.3 Effect of NCS Addition on the Textural Properties of LMP Gels

**Figure 1:**
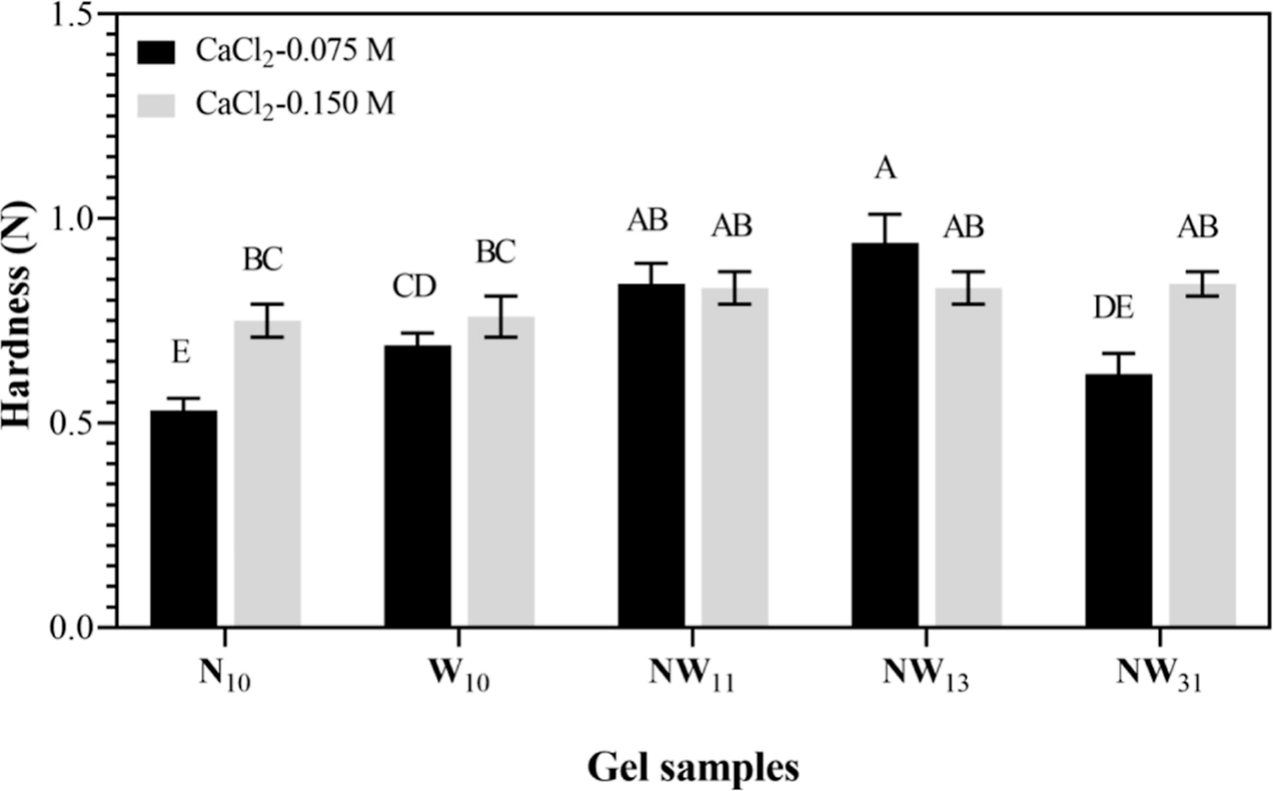
Hardness values of gel samples. Different letters indicate significant difference (*p* < 0.05).

The hardness values of LMP gels formed using NCS, WS, and their mixtures with 0.075 and 0.15 M concentration of CaCl_2_ are shown in Figure 1. The highest hardness value (0.94 N) was observed in the NW_13_ gel prepared at 0.075 M concentration of CaCl_2_, whereas the minimum hardness (0.53 N) was observed in the N_10_ gel prepared by using the solely NCS with 0.075 M concentration of CaCl_2_. A significant increase in hardness was observed with decreasing NCS concentration in gel (*p* < 0.05) at 0.075 M CaCl_2_ for the NW_11_ and NW_13_ gels, which were prepared by replacing 50% and 25% of WS with NCS, respectively. A similar hardness was attained when utilizing mixtures of NCS and WS at 0.15 M concentration of CaCl_2_ (*p* > 0.05). Besides, it was observed that an increase in CaCl_2_ concentration led to a significant increase in hardness of only N_10_ and NW_31_ gels (*p* < 0.05), while for the other gels, this trend was not observed (*p* > 0.05). This was likely due to the additional cross-linking in the gel matrix, but the effect was not as pronounced in samples with lower NCS content.

At 3.5 pH or below, the LMP gelation was facilitated by the hydrostatic interaction and hydrogen bond compared to the high pH (around 7), where more Ca^2+^ is required to facilitate the gelation of LMP due to the increase in the number of sequences of dissociated carboxyl groups available for the Ca^2+^ binding.^15,16,45^ At a low CaCl_2_ concentration, N_10_ gel showed a lower hardness than W_10_ gel. However, the CaCl_2_ concentration was increased to 0.15 M, which was the standard concentration of LMP gel according to Food Chemical Codex (1972);^19^ NCS replacement resulted in similar values in terms of hardness. Additionally, after mixing these two sugars in equal amounts, gels reached maximum hardness, and CaCl_2_ concentration did not show a significant effect (*p* > 0.05).

Replacement of 75% of WS with NCS (NW_31_) at 0.075 M CaCl_2_ concentration resulted in nearly similar values with only the WS-used gel. NCS alone was found insufficient to contribute to the gel hardness. It is very likely that other components, such as minerals and phenolics, did not enhance the strengthening of the junction zones. However, this limitation was overcome by adding small amounts of WS. It is well-known that the formation of complexes between pectin and Ca^2+^ in LMP gels is facilitated by sugar. The stronger water-binding ability of sugar compared to pectin prevents water molecules to form hydrogen bonds with the carboxyl groups of pectin, making it favorable for the formation of hydrogen bonds with Ca^2+^^34^^,^.^46^ Similar results were reported by Wan et al.^47^ where it was shown that the addition of sugar alcohol instead of sucrose decreased the gel strength of LMP gels. They explained the behavior for two reasons. First, sugar alcohols were found to compete with pectin for water, resulting in reduced hydration of pectin molecules to bind calcium. Second, the binding of sugar alcohols to calcium ions interfered with the formation of calcium-pectin complexes. Grosso et al.^46^ mentioned that the sugar and water interaction was the secondary effect, while the competence between the sugar and water for calcium had more importance on gel rigidity for LMP.

**Figure 2:**
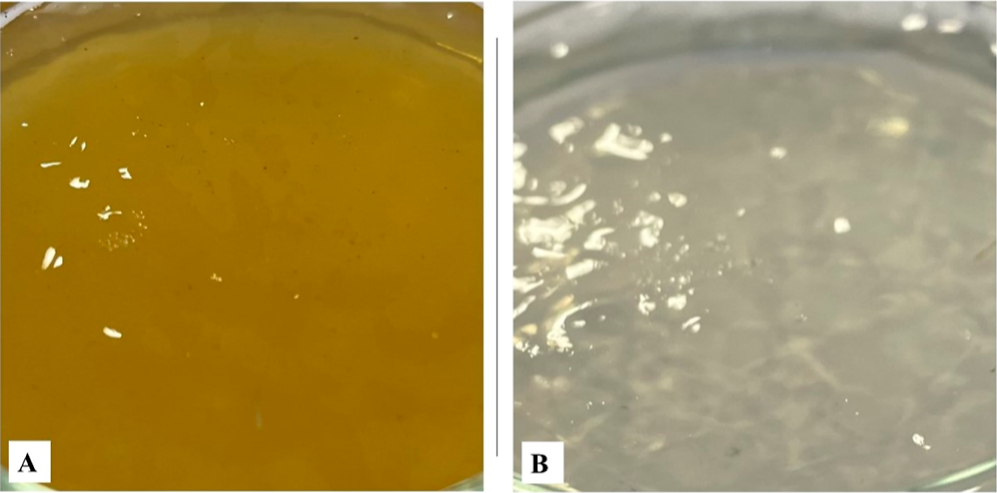
Visual image of gels: (A) N_10_ and (B) W_10_ gel.

NCS contains mainly sucrose (76.55–89.48%) with small contributions of glucose and fructose (3.69–10.5) and other substances such as water, amino acids, minerals, vitamins, and polyphenolic compounds.^10^ These compounds give NCS a natural yellowish-brown color which transferred to the gels. On the contrary, WS is colorless and composed of sucrose with a very high purity (>99.9%).^48^ The color difference between the N_10_ and W_10_ gels can be seen in Figure 2. As it is known, sucrose did not compete with pectin for calcium binding through LMP gel formation.^46^ Thus, it is expected to achieve reduced hardness in NCS-containing gels compared to WS-containing gels due to interference of substances other than sucrose. Although impurities, including naturally occurring calcium within NCS, were expected to enhance gelation and boost hardness, they failed to achieve this objective. Instead, in our cases, these impurities seemed to hinder gelation by competing with both pectin and calcium, ultimately leading to a reduction in the hardness values. If substances other than sucrose bind to pectin, this interaction may cause steric repulsion, hinder the agglomeration of dimers formed after calcium binding, and consequently reduce the rigidity of the junctions in LMP gels. Phenolics, for example, can form hydrogen bonds with pectin molecules and reduce available carboxyl groups to bind Ca^2+^ and may decrease the gel’s texture and strength.^49^ This is another important factor for the gelation of LMP that ionic linkages via calcium bridges occur between two available carboxyl groups from two different pectin chains in close proximity.^39^ In their work,^17^ they obtained similar results—sugar cane molasses-added LMP gels showed low gel strength due to interference of polyphenols and inorganic salts, which compete with calcium for effective junction zones during the gelation process. Thus, mixing NCS with WS could reduce the interference of other substances, resulting in values similar to those of the standard LMP gel. It can be concluded that NCS alone was ineffective in increasing gel hardness, but it can be used as a partial replacement for WS at some concentration without compromising the LMP gel’s strength.

### 3.4 Effect of NCS Addition on the Rheological Properties of LMP Gels

**Figure 3:**
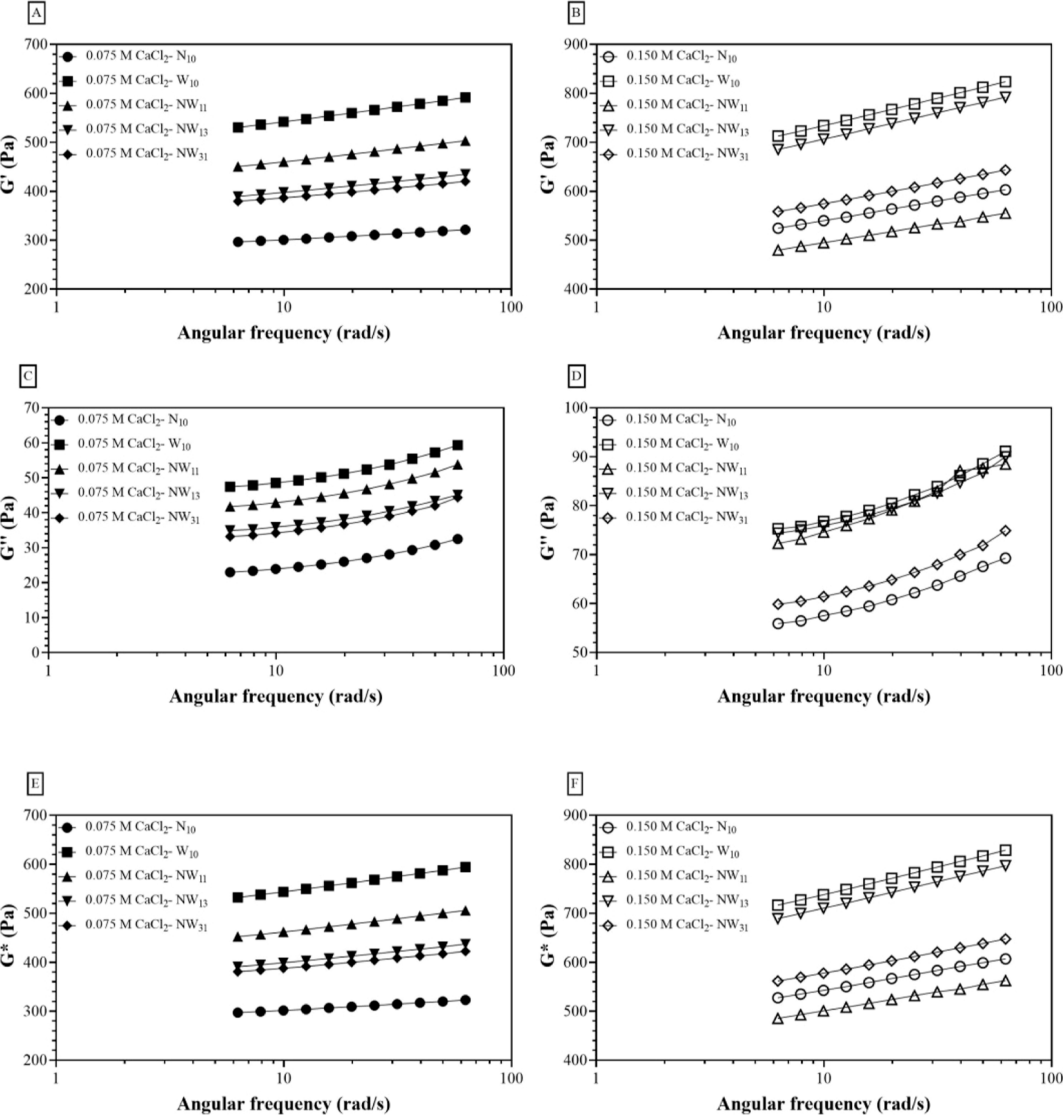
Variation of elastic (*G*′, ▶) and loss (*G*′′, ■) moduli in response to the oscillation frequency of LMP gels added with CaCl_2_ at two concentrations as (a) 0.075 and (b) 0.15 M.

The viscoelastic behavior of gels formed using NCS, WS, and their mixtures with 0.075 and 0.15 M concentrations of CaCl_2_ is shown in Figure 3. The *G*′ values (Figure 3A,B) observed are always higher than *G*″ values (Figure 3C,D), indicating solid-like and elastic gel properties for all samples.^22,50,51^ Besides, all gels remained relatively stable, showing frequency-independent behavior throughout the whole range within measurements. The viscoelastic components (*G*′ and *G*″) of WS were observed to be higher than the NCS at both 0.075 M (Figure 3A,C) and 0.15 M concentration of CaCl2 (Figure 3B,D), whereas the gels prepared by using their mixtures were found in between. In agreement with the hardness results, the *G*′ value of NCS gels showed less solid-like behavior, while WS only showed the greatest hardness along with the highest *G*′ value. At the 0.075 M concentration of CaCl_2_, NW_11_ gel (1:1 ratio of NCS to WS) increased the elasticity toward the value of WS_10_ gel. However, at the 0.15 M concentration of CaCl_2_, the NW_13_ gel (1:3 ratio of NCS to WS) caused a slight reduction in both *G*′ and *G*″ values and behaved similarly with WS_10_ gel. The increasing trend was noted in both *G*′ and *G*″ with the concentration of CaCl_2_, as it is directly related to the increasing cross-linking density.^52,53^ Thus, the ability of NCS to produce hard LMP gels could be promoted with a high CaCl_2_ concentration. It has been stated that sugar enhances the storage modulus of gels.^16^

The power law model parameters are shown in Table 5. The consistency indexes (*k*′ and *k*″) and flow index behaviors (*n*′ and *n*′′) of the rheological parameters (*G*′ and *G*″) were well fitted to the power law model, showing the coefficient of determination (*r*^2^) above 0.98. As expected, the values of *k*′ increase with the concentration of CaCl_2_, indicating that the gels become more viscous as the CaCl_2_ concentration increases. The W_10_ at 0.150 M CaCl_2_ has the highest *k*′ value, suggesting that it has the highest viscosity among the samples.^23^ The *k*′ values appear to be influenced by the ratio of NCS to WS. For example, at 0.075 M CaCl_2_, the *k*′ value for NW_11_ is lower than that of W_10_ but higher than that of N_10_. This suggests that both sugars in a balanced ratio provide a moderate consistency compared to the extremes of pure NCS or WS. The values of n’ are generally less than 1, indicating shear-thinning behavior for all samples.^23,24^ The W_10_ at 0.150 M CaCl_2_ had the highest *n*’ value, suggesting that it has the most pronounced shear-thinning behavior. Similar to *k*′, the elastic modulus *k*″ increases with the concentration of CaCl_2_, indicating stronger gel structures at higher CaCl_2_ concentrations.

**Table 5:** Parameters of Power Law Fitting of Storage Modulus (*G*′), Loss Modulus (*G*″), and Complex Modulus (G) of LMP Gels^a^

sample		*k*′	*n*′	*r*^2^	*k*″	*n*″	*r*^2^	*A*	*Z*	*r*^2^
0.075 M CaCl_2_	N_10_	277 ± 8.63E	0.036 ± 0.004d	0.9992	17.0 ± 0.56C	0.150 ± 0.003a	0.9773	277.7 ± 8.60E	27.85 ± 2.53a	0.9989
	W_10_	486 ± 74.7BCD	0.048 ± 0.004 cd	0.9999	38.9 ± 7.46ABC	0.098 ± 0.011 cd	0.9768	488 ± 75.1BCD	21.00 ± 1.77bcd	0.9999
	NW_11_	412 ± 49.8CDE	0.049 ± 0.003bc	0.9999	33.4 ± 4.04ABC	0.110 ± 0.009bc	0.9806	413 ± 49.9CDE	20.38 ± 0.95bcde	0.9998
	NW_13_	356 ± 50.7CDE	0.048 ± 0.008 cd	1	27.9 ± 6.32BC	0.113 ± 0.014bc	0.9762	357 ± 50.9CDE	21.26 ± 3.42bc	0.9999
	NW_31_	349 ± 38.2DE	0.045 ± 0.003 cd	0.9994	25.8 ± 3.68BC	0.124 ± 0.005b	0.9769	350 ± 38.4DE	22.24 ± 1.04b	0.9992
0.150 M CaCl_2_	N_10_	469 ± 48.1BCD	0.061 ± 0.004ab	0.9999	46.2 ± 7.78ABC	0.096 ± 0.007 cd	0.9889	471 ± 48.7BCD	16.39 ± 0.96cde	0.9998
	W_10_	635 ± 52.8A	0.064 ± 0.004a	0.9999	63.4 ± 4.07A	0.084 ± 0.005d	0.9776	638 ± 52.9A	15.77 ± 0.79e	0.9999
	NW_11_	428 ± 72.2CD	0.063 ± 0.009a	1	60.0 ± 29.0A	0.095 ± 0.01 cd	0.9935	433 ± 75.4BCD	15.84 ± 1.86e	1
	NW_13_	611 ± 23.4AB	0.064 ± 0.002a	0.9998	63.1 ± 1.88A	0.081 ± 0.005d	0.9847	614 ± 23.4AB	15.79 ± 0.40e	0.9999
	NW_31_	499 ± 53.0ABC	0.062 ± 0.003ab	1	49.4 ± 6.33AB	0.096 ± 0.005 cd	0.9825	501 ± 53.3ABC	16.15 ± 0.72de	1

a *Values are shown as mean ± standard deviation. Values within the same analysis indicated by different letters are significantly different (*p* ≤ 0.05).

The ratio of NCS to WS seemed to affect *k*″, with mixed ratios (e.g., NW_11_ and NW_13_) showing intermediate values compared to the pure samples (N_10_ and W_10_). This indicated that the sugar composition influenced the gel structure’s elasticity. The W_10_ at 0.150 M CaCl_2_ had the highest *k*″ value, consistent with its high viscosity. The values of *n*″ are generally less than 0.15, indicating that the elastic behavior dominates over the viscous behavior in these gels. There is no clear trend in *n*′′ with ratios of NCS to WS or the concentration of CaCl_2_ similar to *n*′ values. The *n*′ and *n*″ values are much lower than those reported albumin-LMP (amidated) gels (0.11–0.56)^54^ and xanthan gum-soy protein gels (0.12–0.16).^55^ It has been stated in a report^56^ that the flow behavior indexes above 0 suggest noncovalent physical cross-linking of the gel network. Thus, it is the confirmation of the stabilization of gel networks by physical interactions, such as hydrogen bonds, hydrophobic interactions, or electrostatic forces rather than covalent chemical bonds.

Further, the complex modulus (G*) over the angular frequency data was fitted, and parameters *A* and *z* were calculated. The highest value of *A* was noted in W_10_ gel at a higher concentration of CaCl2, while the value of *z* was highest in N10 gel at a lower concentration of CaCl_2_. The strength of the gel increases with a higher value of *A*, while the higher value of *z* indicates a greater number of interactions between strands in the three-dimensional (3D) network.^23−25^ The W_10_ gel was found to be the strongest even with a smaller number of interactions, which means that the interactions between the three-dimensional network of the gel were quite strong. On the other hand, the N_10_ gel was found to have a greater number of interactions but weaker than the W_10_ gel. In the case of NW13 gel, similar values of *A* and *z* were obtained as in W_10_ gel, indicating that the same LMP strength can be achieved by replacing the 25% WS with NCS. Usually, adding sucrose at a constant concentration of pectin increases gel strength, as it provides an additional OH group for the stabilization of the junction zone in a 3D network system of gel.^57^ The extra OH group creates the hydrogen binding to entrap the freer water molecules in a 3D gel network system.^58^

In the current study, the results suggested that the addition of sucrose in the form of NCS might provide the less OH group for hydrogen bond as compared to WS as the NCS contains 10–23% less sucrose concentration than WS.^10,59^ Usually, adding sucrose at a constant concentration of pectin increases gel strength, as it provides an additional OH group for the stabilization of the junction zone in the 3D gel network system.^57^ The extra OH group creates the hydrogen binding to entrap the free water molecules in a 3D gel network system.^58^ Overall, the rheological properties of LMP gels were influenced by the ratio of NCS to WS and the CaCl_2_ concentration. Mixed ratios of NCS to WS result in intermediate rheological properties compared to the pure samples, indicating that the sugar composition plays a role in determining the gel’s consistency, elasticity, and viscoelastic behavior.

### 3.5 Effect of NCS Addition on the *T*_2_ (Spin–Spin) Relaxation Properties of LMP Gels

The physical state of water in the polymeric gel network can be observed through *T*_2_ relaxation times with NMR experiments.^60^ Water is an essential part of the gel. The gel forms a three-dimensional network via the cross-linkage of polymer chains that also entraps the water molecules in it, which gives it a unique rigid structure.^61−63^ Therefore, the gel’s strength depends on the gel’s water-holding capacity.^64^ Moreover, the gel’s shelf life depends on the water with the holding capacity of gels,^65^ as the microbial growth is sensitive to moisture content.^66^ The gels which contain free water molecules are more susceptible to microbial growth, causing a decrease in the shelf life of gels.^67^ By manipulation of the water mobility or state, it is possible to control the microbiota composition, effectively reducing the risk of spoilage and pathogenic microorganism growth. *T*_2_ relaxation time depends on the water-holding capacity of the gel and the concentration of pectin.^64^ Since, in this study, the constant concentration of pectin was used, *T*_2_ relaxation time was associated with the water-holding capacity of the gel. In another study, a high correlation was observed between the water-holding capacity and the *T*_2_ relaxation time of the gel.^68^

In the absence of CaCl_2_, *T*_2_ relaxation times of WS, pectin, and water mixture were measured as 295.6 ms, while it was 170.4 ms for NCS, pectin, and water mixture. This could indicate that NCS might have a higher water-binding capacity or a more complex water structure, possibly due to impurities or components in NCS than WS. However, *T*_2_ relaxation times decreased significantly with the addition of CaCl_2_ (Table 6) (*p* < 0.05) by 1.1-fold for NCS at both concentrations and 1.6 and 1.7-fold for WS at 0.075 and 0.15 M concentrations, respectively. Gelation creates a network, traps water, and decreases the mobility of water protons, which decreases the relaxation times. The decrease in *T*_2_ values for NCS and WS after the addition of CaCl_2_ indicated the structural and textural changes in the gels that altered the NMR relaxation times, and on a molecular level, it was shown that NCS might have inherently different gelation properties compared to WS. In addition, doubling in CaCl2 concentration did not significantly change the *T*_2_ values for NCS (*p* > 0.05) but decreased the *T*_2_ value for WS to 1.7, suggesting that NCS might exhibit a certain level of resistance or saturation in response to increasing calcium ions.

**Table 6:** Monoexponential *T*_2_ Values of the LMP Gels^a^

sample ID	*T*_2_ (ms)	
	0.075 M CaCl_2_	0.15 M CaCl_2_
N_10_	156.9 ± 13.7CD	155.7 ± 10.4CD
W_10_	184.3 ± 8.15A	174.1 ± 9.57AB
NW_11_	162.6 ± 7.07BC	139.8 ± 9.52E
NW_13_	163.8 ± 12.8BC	159.1 ± 9.17CD
NW_31_	160.0 ± 4.47BCD	147.7 ± 3.43DE

a *Values are shown as mean ± standard deviation. Values within the same analysis indicated by different letters are significantly different (*p* ≤ 0.05).

Among the *T*_2_ values of gels prepared shown in Table 6, at a low CaCl_2_ concentration, the replacement of NCS significantly decreased the *T*_2_ values of gels (*p* < 0.05), but increasing the NCS concentration within the gel matrix did not affect the results (*p* > 0.05). Conversely, at a 0.15 M CaCl_2_ concentration, the decrease in *T*_2_ values was found to be significant with NCS addition, and reduction was observed with the increase of concentration of NCS in gels and reached the lowest of 139.8 ms for the NW_11_ sample. The lowest *T*_2_ values indicated the strongest gel, while in the case of hardness, the highest hardness value was observed in NW_13_ gel prepared at the 0.075 M concentration of CaCl_2_. However, a similar pattern was observed in the hardness as a significant decrease in hardness was observed with the increasing NCS concentration in gel (*p* < 0.05) until 50% replacement of NCS with WS at both concentrations of CaCl_2_. The viscoelastic behavior of studied gels did not follow a similar pattern, as it was found to be highest in the W_10_ gels. The thickness of NW_11_, NW_13_, and NW_31_ was found between the W_10_ and N_10_ gels. The lower *T*_2_ times of NCS gels compared to those of WS gels confirmed that it could compete with pectin for water and showed higher water binding ability than WS to form a hard gel. Besides, *T*_2_ relaxation time was observed to be correlated with the higher storage modulus (*G*′)^69^ and hardness value of the gels.^64^ The shorter *T*_2_ relaxation time indicated the thicker and stronger gel due to constrained water mobility, while the longer *T*_2_ relaxation time indicated the mobility of water molecules. Thus, in this study, the decreases in both hardness and viscosity of NCS gels were observed as compared to the WS gels due to creating a more open or less cross-linked gel structure that influenced *T*_2_ relaxation time.

The impurities within NCS could also influence the *T*_2_ relaxation times of gels, which can lead to increased relaxation rates of nearby protons if they contain unbound hydrogen groups. However, these effects can be very complex and depend on several factors, such as chemical structure, concentration within, and interaction with the surroundings. Besides, a decrease in the Brix value with the addition of NCS, indicating a lower concentration of solutes like sugars or other compounds in the gel, can lead to longer *T*_2_ relaxation times. This is because the absence of solutes, especially sugars, can lead to less frequent interaction with water and tends to increase *T*_2_ relaxation times. Also, changes in the pH toward high values observed with NCS can lead to longer *T*_2_ relaxation times due to alkaline substances within NCS influencing the ionization state of molecules and functional groups in the solution.

### 3.6 Effect of NCS Addition on the Self-Diffusion Coefficient Measurements of LMP Gels

The NMR diffusion analysis is frequently performed with pulsed field-gradient spin echo (PEG-SE). It has been used to investigate molecular folding, agglomeration, and steric interactions in food materials.^70^ It can also be used to measure the self-diffusion coefficient of free water molecules in complex systems to estimate the movement of the free water molecules in the gel. The diffusion coefficient has a direct relationship with the movement of the water molecules. If water molecules are present in bound form or encounter physical barriers, the values of diffusion coefficient decrease, whereas the values of diffusion coefficient increase if water can move more freely.^71^ The water molecules bind with pectin and sugar in the presence of Ca^2+^ ions during the gelation process in LMP, restricting the free movement of water molecules.^72^ The decrease in the free motion of water molecules indicated that the water molecules diffused to LMP gels and decreased the diffusion coefficient. The self-diffusion coefficient analysis provides valuable insights into the microstructure of gels.^73^

**Figure 4:**
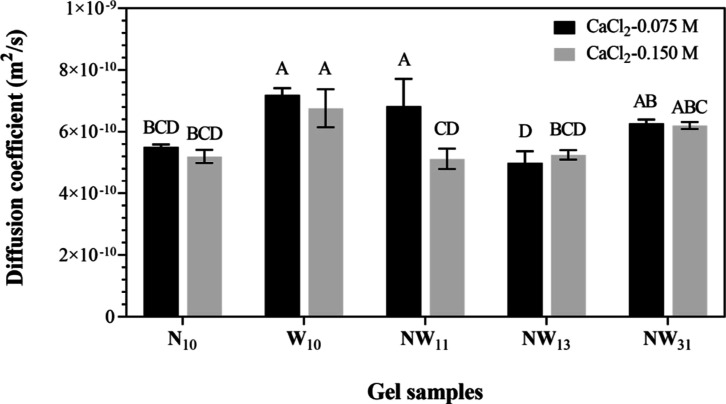
Diffusion value of LMP gels. Different letters indicate significant difference (*p* < 0.05).

The self-diffusion results of LMP gels are listed in Figure 4. The diffusion coefficient values of gels were observed in the range of 4.99 × 10^–10^–7.19 × 10^–10^ m^2^s^–1^. The self-diffusion coefficient decreased with the addition of NCS and increased with CaCl_2_ concentration significantly (*p* < 0.05). Similarly, in the case of rheological study, the thickness of gels was decreased with the addition of NCS and increased with the increase in CaCl_2_ concentration. At the 0.15 M CaCl_2_ concentration, NCS caused minor or insignificant differences (*p* > 0.05). However, at the 0.075 M CaCl_2_ concentration, as the substitution of WS with NCS increased from 0% to 100% in the gel formulations, self-diffusion coefficients exhibited a progressive rise, demonstrating a direct relationship between WS concentration and the enhanced self-diffusion coefficient. Surprisingly, at the 25% NCS concentration (NW_13_), there was a notable and sudden reduction in self-diffusion, showing a similar value to N_10_ as a similar pattern was noted in the case of hardness of the gels. This situation suggested a critical threshold in the influence of NCS on water and solute mobility within the LMP gels. They were also correlated with the *T*_2_ values that the water binding ability of NCS was higher than that of WS, which restricted the mobility of water molecules within the gel and showed a low self-diffusion coefficient.

The highest diffusion coefficient values of 7.19 × 10^–10^ m^2^s^–1^ at the 0.075 M concentration of CaCl_2_ were observed in the W_10_ sample. The high self-diffusion coefficient for WS corresponded to a softer gel that allowed water molecules to move freely within the gel matrix while reducing the overall hardness. In fact, after replacement with NCS, indicated by the lower self-diffusion coefficient due to molecular confinement or restricted mobility, it resulted in a more rigid gel (NW_13_). The presence of solutes other than sugars in NCS could impact the self-diffusion coefficient differently than WS, and their interaction may be a critical factor in determining the gel properties for NCS in LMP gel. Thus, the changes in the self-diffusion coefficient can be attributed to alterations in the gel microstructure, the influence of calcium in strengthening the gel matrix, and the impact of NCS components on water binding and molecule mobility within the gel. The hardness of the studied gels was increased with the replacement of NCS with WS from the W_10_ gels while the thickness remained below the W_10_ gels. The replacement of 25% NCS with WS achieved an almost similar thickness as pure W_10_ gels but was still noted below the thickness of W_10_ gels. Thus, these findings highlight the complex nature of LMP gelation and the versatile effects of different components on gel properties and behavior.

In this study, NCS was used to produce the LMP gel. Notably, it has been possible to partially replace the WS with NCS in the gel formulation to achieve comparable rheological characteristics. The complete replacement of WS with NCS reduced the hardness of the gel because of the interference of the other compounds present in NCS. Even though these compounds are the main reason for adding health benefits to NCS gel, they also compete with both the pectin and calcium ions and reduce the rigidity of the junctions in LMP gel formation. Both NMR and MRI techniques were shown to be valuable tools employed for the analysis of molecular and macroscopic properties of LMP gels and can be used in complementary ways to gain a comprehensive understanding of gel behavior. It was found that the best NCS/WS ratio used to make the stronger LMP gel was 1:3, within the scope of our study. Therefore, it can be inferred that partial substitution of WS with NCS in the formation of LMP gels may be feasible, while preserving the gel’s texture and simultaneously utilizing its distinct health-enhancing properties. Further studies are needed to characterize the NCS gelation behavior.
